# Body form and body motion processing are dissociable in the visual pathways

**DOI:** 10.3389/fpsyg.2014.00767

**Published:** 2014-07-21

**Authors:** Paddy D. Ross

**Affiliations:** Institute of Neuroscience and Psychology, University of GlasgowGlasgow, UK

**Keywords:** action perception, double dissociation, form and motion, EBA, pSTS

The human body is a salient communicator of social information, such as aggression, intention, and affective state. Although brain areas specializing in human body recognition have been identified, little is understood about how these areas interact and contribute to the different components of body recognition (see de Gelder et al., 2010).

One model of action discrimination proposes that signals conveyed by the body are processed along two parallel pathways in the brain, a dorsal pathway analyzing body motion signals and a ventral pathway analyzing body form signals (Thompson and Baccus, 2012; Miller and Saygin, 2013) (see Figure 1A).

**Figure 1:**
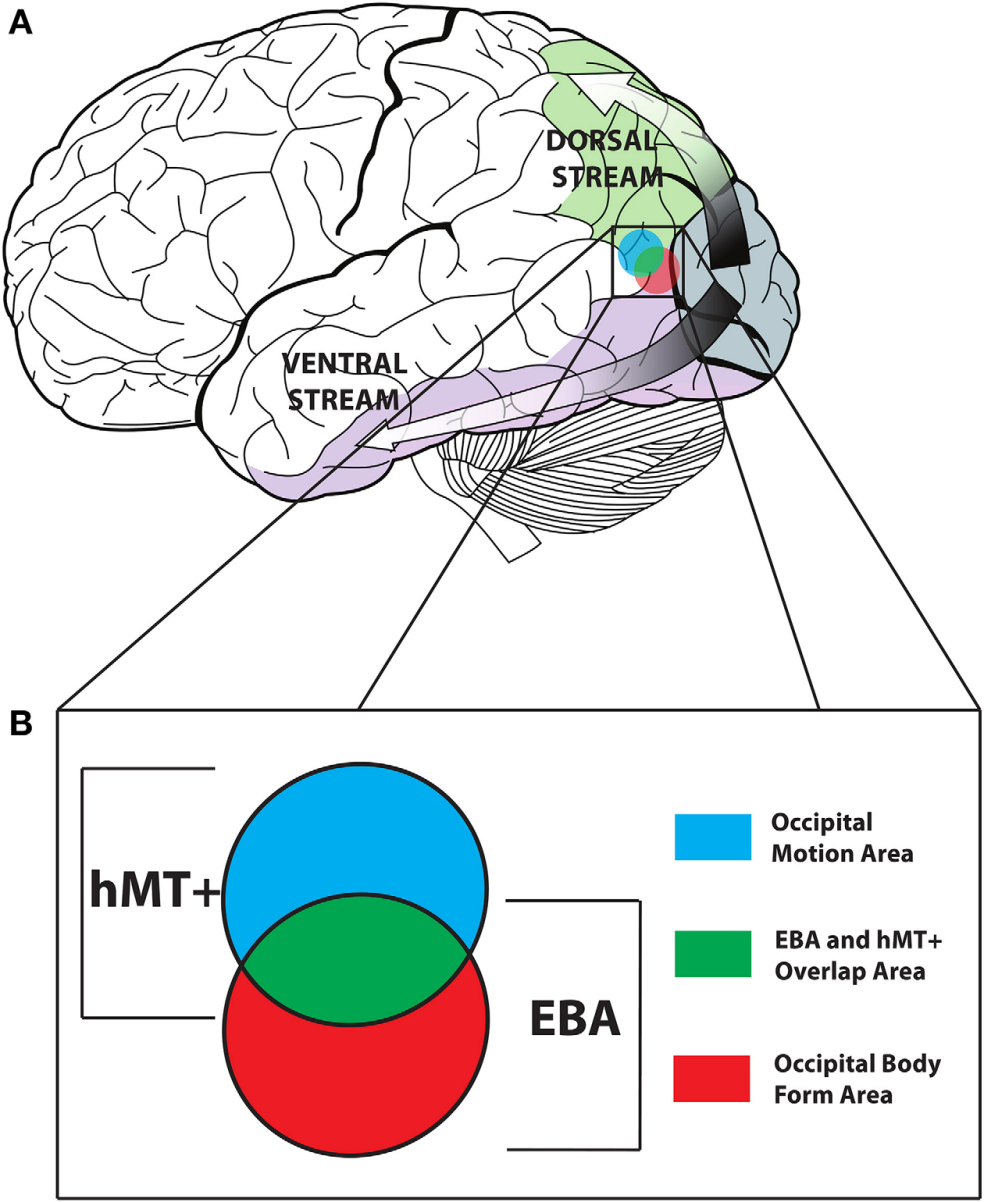
**(A)** The dorsal (motion) and ventral (form) pathways of the visual system. **(B)** The overlap of EBA and hMT+ is presented in Green. The Occipital Motion Area (formally hMT+^*^) is shown in Blue, and the Occipital Body Form Area (formally EBA^*^) is shown in Red. Adapted from an image created by Lokal_Profil, CC-BY-SA-3.0.

The posterior superior temporal sulcus (pSTS) is hypothesized to be involved in processing body motion cues, while the extra-striate body area (EBA) and fusiform body area (FBA) are hypothesized to be involved in processing body form cues (Peelen et al., 2006; Grossman et al., 2010; Grosbras et al., 2012). However, dissociating these two pathways remains a contentious issue in the body recognition literature. Firstly, there is substantial cortical overlap between EBA and the human motion complex (hMT+). Secondly, body form and motion are closely linked and integrated; body motion can give clues about body form such as gender, identity and emotion (Pollick et al., 2005; Ross et al., 2012); conversely body posture conveys information about intended movement (Cazzato et al., 2012). A recent study conducted by Vangeneugden et al. published in *The Journal of Neuroscience*, addressed these issues in a novel way (Vangeneugden et al., 2014).

The purpose of their investigation was to explore whether the neural mechanisms that process body form and motion cues are dissociable. The authors used a combination of functional magnetic resonance imaging (fMRI), psychophysics, and transcranial magnetic stimulation (TMS) to address this question. First, they tackled the issue of EBA/hMT+ overlap by localizing each region of interest (ROI) independently. Voxels selective for both bodies and motion in both ROIs (and so would be included in both EBA and hMT+) were then excluded from further analysis (at least from the fMRI portion of the study). This effectively created two new functional regions which the authors labeled EBA^*^ and hMT+^*^. To look for evidence of a double dissociation in body form and motion processing, the authors used fMRI and multivoxel pattern analysis (MVPA) to ask whether multivoxel patterns in EBA^*^ and pSTS selectively carried information about the form and motion of whole body point-light display walkers (PLW). The MVPA results yielded a stark double dissociation: the EBA^*^ carried information about the PLWs body posture but not motion, while the pSTS carried motion direction information, but nobody posture information.

Next, the authors investigated whether the representations uncovered in the two ROIs causally contributed to behavioral discriminations of body form and motion. To address this question they created a novel set of stimuli of PLWs consisting of ellipses with variable alignments. This allowed the authors to manipulate form information while leaving the movement trajectories unaffected, and *vice-versa*. They found that elliptical misalignment (form manipulation) had a stronger effect on a facing orientation task than on a walking direction task, while stimuli duration (motion manipulation) gave the opposite effect. Thereby these results strongly indicate that body form and body motion processing rely on separate visual cues.

Finally, the authors used the same psychophysical tasks in a repetitive TMS experiment, during which they manipulated neural activity in either the EBA or pSTS. They observed a double dissociation, with TMS over EBA disrupting performance in the form discrimination task significantly more than TMS over pSTS, and *vice-versa* for the motion discrimination task.

These results provide converging evidence that the perception of body form and motion relies on distinct functional and neural pathways, in keeping with the parallel processing pathways model of body action perception. However, there are a few caveats that are worth reiterating.

In separating the EBA and hMT+ into two regions containing only voxels that were selective to bodies and motion respectively (EBA^*^ and hMT+^*^), Vangeneugden et al. (2014) have set a standard from which future work into the dissociation of form and motion processing in the visual system should adhere to. In doing so, however, they have effectively made comparison with other papers exploring the EBA and hMT+ impossible. EBA^*^ and hMT+^*^ should be treated as distinct areas in their own right in future work, perhaps being renamed to avoid confusion [The Occipital Body Form Area (OBFA) and Occipital Motion Area (OMA) for example, see Figure 1B]. This is significant because, as Vangeneugden et al. (2014) duly acknowledge, the occipital area that they target in the TMS portion of their study also most likely contained hMT+. This could have led to disruption of motion processing in hMT+, although the authors give several reasons why hMT+ stimulation may not lead to impaired biological motion discriminations.

The main issue is that one could argue that the areas in which the double dissociations were observed in the fMRI and TMS portions of Vangeneugden et al. (2014) study were not the same. This is important, as if future studies are to build upon this work effectively, they must make the distinction between EBA and hMT+, and EBA^*^ and hMT+^*^.

Overall, the results of the Vangeneugden et al. (2014) study offer a valuable insight into whether functional and neural body perception mechanisms are dissociable. Their evidence supports a parallel pathways model of body action perception. Methodologically they also set a new precedent for form and motion dissociation work by first separating the EBA and hMT+. In terms of building for the future, the on-going challenge will be to explore other modulatory networks to build a comprehensive model of body perception. This is especially important because, as the authors point out, distinct clinical profiles such as autism spectrum disorders and eating disorders have been associated with EBA and pSTS abnormalities. So, a more complete body recognition model will shed new light on these clinical findings, and ultimately provide a rich analytical framework for investigating other social cognitive disorders.

## Conflict of interest statement

The author declares that the research was conducted in the absence of any commercial or financial relationships that could be construed as a potential conflict of interest.
